# Enabling Canadian Physician Wellness in the Age of Digital Innovation: What Do We Need to Succeed?

**DOI:** 10.1055/a-2628-1323

**Published:** 2025-11-07

**Authors:** Tania Tajirian, Brian Lo, Adam Tasca, Brittany Poynter, Gwyneth Zai, Gillian Strudwick, Chandi Chandrasena, Karim Jessa, Phil Shin, Julie Maggi, Uzma Haider, Andrew Pinfold, Ashley P. Miller, Ashley Chisholm, Dawn Lake, Damian Jankowicz, Sanjeev Sockalingam

**Affiliations:** 1 Centre for Addiction and Mental Health, Toronto, Ontario, Canada; 2 University of Toronto, Toronto, Ontario, Canada; 3 Unity Health, Toronto, Ontario, Canada; 4 OntarioMD, Toronto, Ontario, Canada; 5 The Hospital for Sick Children (SickKids), Toronto, Ontario, Canada; 6 Vancouver Physician Staff Association, Vancouver, British Columbia, Canada; 7 Nova Scotia Health, Halifax, Nova Scotia, Canada; 8 IWK Health, Halifax, Nova Scotia, Canada; 9 Canadian Medical Association, Toronto, Ontario, Canada; 10 Doctors of BC, Vancouver, British Columbia, Canada

**Keywords:** burnout, physician wellness, electronic health records, digital health

## Abstract

**Background:**

Digital health tools, such as artificial intelligence scribes, offer significant potential to alleviate physician burnout and reduce administrative burdens associated with electronic health records. Despite their promise, Canadian health care organizations face challenges in establishing cohesive strategies for their effective implementation and evaluation.

**Objectives:**

This paper explores actionable, organizational strategies to enhance physician wellness through digital health tools. It examines systemic barriers, promising practices, and infrastructure needs, culminating in five key recommendations for sustainable adoption.

**Methods:**

An environmental scan assessed digital health initiatives across Canada, incorporating case studies from wellness committees, advisory councils, and physician-led programs. National surveys and evaluation frameworks were reviewed to identify barriers, facilitators, and outcomes.

**Results:**

Findings highlight challenges such as insufficient training and funding, fragmented governance and policies, and varied accessibility to digital tools. Promising initiatives demonstrated reduced documentation burdens, improved physician satisfaction, and streamlined workflows. Successful strategies included forming advisory committees, developing governance frameworks, and implementing standardized training programs. However, systemic barriers, including funding constraints and resistance to change, persist and require targeted interventions.

**Conclusion:**

The responsible adoption of digital health tools in Canadian health care demands robust governance, equitable funding, and standardized toolkits tailored to diverse settings. Active physician engagement and comprehensive training programs are essential to overcoming systemic challenges and fostering sustainable improvements in physician wellness and health care system efficiency.

## Background and Significance

Physician burnout related to the use of digital tools, which includes electronic health records (EHR), artificial intelligence (AI)-based solutions, speech recognition technologies, and other tools continues to be a critical challenge jeopardizing the sustainability of health human resources and health care systems in Canada. 1 2 3 4 With over 61% of Canadian physicians reporting burnout in 2021, there needs to be systemic solutions to address the root causes including administration burden, poor usability and workflow integration of digital tools, as well as other causes that have been well documented in the literature. 5 6 7 8 For example, the 25 × 5 Task Force led by the American Medical Informatics Association highlighted that the excess effort required to complete documentation has led to poor satisfaction with EHR systems. 9 10 In this article, we outline the effect of digital tools on physician wellness and discuss recommendations for health care organizations across Canada to improve their readiness and adoption of these solutions.

## Methods

For the purpose of this study, we utilized a thorough review conducted by the Canadian Medical Association (CMA) of contributors to physician burnout including administrative burden, lack of work-life balance, and poor social well-being. 5 We also looked at the results of the 2024 National Survey of Canadian Physicians commissioned by the CMA and Canada Health Infoway, which detailed a link between physician burnout and time spent on EHRs, with physicians reporting an average of 107 minutes per day outside their typical workday. 11 In particular, we utilized results from respondents identifying the barriers to optimal use of digital health technologies. To develop recommendation, we considered the lack of integration, multiple logins, and the nonintuitive nature of clinical information systems. 11 We further refined our recommendations by considering the results of CMA's administrative burden working group that recommended (1) championing interoperability through legislation; (2) eliminating sick notes; (3) addressing federal and national forms; and (4) building a position on AI and administrative burden. 12

In addition to these initiatives, we consulted with the Ontario Medical Association (OMA) who highlighted that burnout is mainly caused by health system issues. We utilized the findings from their burnout task force, which suggested that effective ways to reduce the instances of burnout requires system-level solutions linked to the Quadruple Aim of health, a framework used to ensure that a health care system is sustainable by “improving the patient and caregiver experience; improving the health of populations; reducing the per capita cost of health care; and improving the work–life of providers.” 13 Additionally, as part of this work the OMA proposed five evidence-based system-level solutions including (1) streamlining (e.g., limiting redundant documentation, reducing the complexity of documentation, reducing the number of required clicks and keystrokes, etc.) and reducing documentation and administrative work; (2) fair and equitable compensation for work and time; (3) better work–life balance arrangements; (4) implementation of physician wellness initiatives at health care organizations; and (5) creating seamless integration of different digital health tools without the need to learn every tool. 13 These solutions greatly influenced our considerations when developing our recommendations. Lastly, we utilized the 2023 Doctors of BC Health Authority Engagement Survey, which reports that only 43% of physicians felt they could reasonably balance work demands with personal life, reflecting the ongoing stress and pressure in the health care system and furthered our national recommendations. 14

## Results

From the case studies we analyzed, it was determined that as physicians begin to use digital health solutions, such as generative AI tools to address EHR and documentation burden issues, 15 16 there is an urgent need for health care systems to provide the infrastructure to enable the value of digital tools supporting physician wellness. The 2024 survey by the CMA and Canada Health Infoway found that 7% of physicians are now using AI in their practice to support patient care, which is up from 2% in 2021. 17 Without best practices and policies in place, adoption of these digital solutions may be low and worsen physician burnout, resulting in negative effects on patient care and safety. 18 To address this gap, efforts have been made to bring a collaborative approach to addressing the issue. A Canadian example of this comes from the Toronto Academic Health Sciences Network; medical leadership in this network created a subcommittee to provide direction to leadership on matters related to wellness. The subcommittee created a digital health task force to better understand the initiatives, gaps, and opportunities surrounding the adoption of digital tools for physician wellness. 19

OntarioMD led an AI scribe evaluation study with community partners, developing a framework to assess this emerging technology from a community health perspective. AI scribes refer to digital tools that utilize AI to produce medical documentation through summarization of health records and/or listening to consultations between patients and providers to automatically write a clinical note. 20 The study aimed to identify key opportunities, gaps, and challenges. Evaluation criteria included usability, functionality, liability, and privacy, ensuring AI scribes meet physicians' needs while prioritizing patient safety and data security. 20

In British Columbia, the Provincial Health Services Authority has been actively working to improve the digital infrastructure to support AI-driven tools, like AI scribes, with Doctors of BC. However, results also show that ensuring AI scribes meet physicians' needs is a critical factor. 21 While some progress is being made, many physicians still report challenges in having meaningful input into the digital tools affecting their practices. 14

### How are Digital Health Tools Being Explored in the Context of Physician Wellness?

Various digital solutions are currently being used by Canadian health care organizations including AI-related tools for physician wellness. 22 For example, the adoption of digital tools is advancing in larger urban health authorities in BC, such as Vancouver Coastal Health; however, rural areas show significantly lower satisfaction and engagement scores, indicating that the benefits of digital health tools are not evenly distributed across the province or country. 14

Given the novelty of these tools and the lack of guidance and best practices, there remains significant heterogeneity in the strategies taken across the country for the implementation, use, and evaluation of digital tools within health systems. 23 Our environmental scan identified previous work conducted by the TASHN Digital Task Force. This Task Force suggested that three main types of initiatives have been deployed amongst academic health care organizations in Ontario. The first group of initiatives is the development of committees that have a mandate of addressing digital health tools issues. For example, one organization identified the importance of having a Digital Quality Committee. In this committee, frontline clinicians and the information technology staff bring forward any issues that relate to the use of the EHR. By discussing these issues, it is known it can result in improved adoption and meaningful use of digital health tools for clinicians. 19 Another group of initiatives includes those that focus on the optimization of digital health tools. These programs often focus on identifying bottlenecks and barriers in finding ways to improve digital workflows. 24 The last area of focus of these initiatives is the integration of digital tools through physician education and training. 21 Given that clinician training is often raised as a key organizational issue, 25 many approaches have been developed to support ongoing training. For example, one organization developed EHR chat groups between physicians and clinical application teams, encouraging physicians to “drop-in” and discuss any EHR or digital health tools issues that they encounter in their daily practice. This initiative has established dedicated time to discuss these issues and has resulted in better departmental collaborations. 10 Several organizations also host “boot camps” or review sessions to focus on delivering additional “at the elbow” support to improve the use, customization, and adoption of various EHR functionalities and digital tools. Postsecondary medical institutions have also explored the delivery of these initiatives in their entry-to-practice curriculum to educate students before entering the field. 26 Collectively, these initiatives can serve as another approach for engaging frontline physicians in evolving their use of digital tools.

### How do Governance Structures Play a Role in Digital Health Wellness Initiatives?

An important area of digital health is governance structures and how organizations manage requests for changes in digital systems and innovations that affect clinicians' workflows. While most Canadian organizations have governance processes and policies related to digital tools such as the EHR, few organizations have robust structures that support continuous engagement with frontline clinicians. One example is the establishment of advisory councils, which typically involve bringing together many providers to discuss changes submitted by the members. However, the lack of appropriate structures for such groups can lead to variable engagement and inadequate decision-making authority and resourcing for implementing changes. Some organizations also use a provider advisory council that focuses on departmental representation on EHR-related issues. For instance, in one organization, as their EHR system evolved, the advisory table transformed into a collaborative forum for continuously developing digital tools, including AI solutions, to enhance the use of the EHR within the organization. Finally, several organizations emphasized the significance of physician champions on leadership committees for digital health tools initiatives. A few organizations also noted the role of these physician champions in advocating for digital health and AI-related decisions within the broader local, regional, and national quality and wellness committees that can affect system changes.

For example, Doctors of BC has established an Administrative Burdens Working Group to address the significant issues physicians face regarding excessive paperwork and documentation, which contribute heavily to burnout. One key project within the working group involves collaboration with WorkSafeBC, an organization that leads workplace safety in BC through consultation and education with employees about safe work practices, to minimize required repetitive forms and streamline the documentation process for injured patients, making it easier for physicians to complete these tasks efficiently. 27

Based on these findings, we outline five recommendations for Canadian health care organizations to improve their readiness to enable the value of digital health, including AI tools ( Table 1 ). Although these recommendations are intended for Canadian health care organizations, we anticipate that these findings will be translatable as other countries implement digital tools within their health care systems. These recommendations focus on enhancing digital health through both organizational and national strategies. At the organizational level, we recommend the establishment of digital health advisory committees and the implementation of robust governance structures to improve physician engagement. At the national level, we recommend the development of national funding strategies to ensure sustainability of these initiatives. Additionally, there is a need for the creation of a standardized digital health toolkit for health care settings and comprehensive digital health curriculum to train both new and existing physicians.

**Table 1:** Key recommendations for enabling the value of digital health including artificial intelligence-related wellness initiatives in Canadian health care organizations

Organizational recommendations
1. Formation of Digital Health Advisory Committees: establish dedicated committees to drive discussions on digital health tools including AI-related initiatives, ensuring alignment with clinical needs and advancements in health care technology 2. Creation of robust governance structures: implement comprehensive governance frameworks to actively engage frontline physicians, enhancing accountability, transparency, and streamlined communication across all levels of care
**National recommendations**
1. Establishment of National Funding Strategies for Digital Health Initiatives: secure sustainable funding mechanisms to support digital health and AI projects, promoting consistent and equitable access to resources and engagement across the health care sector 2. Development of national standardized toolkit for health care organizations: create a versatile, standardized toolkit adaptable to various health care settings—primary care, community care, and hospitals—to promote consistent digital health practices nationwide 3. Implementation of a comprehensive digital curriculum: design an inclusive digital curriculum to equip the next generation of physicians with digital health competencies and provide ongoing education for current practitioners, fostering continuous adaptation to evolving technologies

Abbreviation: AI, artificial intelligence.

## Discussion

### What are the Current Challenges and Priorities for Digital Health Wellness Initiatives?

The environmental scan highlighted several themes that are key areas of focus for digital health wellness initiatives in Canada. These include (1) the need for dedicated resourcing; (2) the use of data and analytics; (3) delivery of standardized clinical digital templates; (4) workflow optimization mechanisms; (5) the role of generative AI tools (e.g., AI scribes); and (6) importance of interoperability and exchange of health information.

It is evident that several barriers continue to hinder the full adoption of digital tools initiatives in Canada. Foremost, there is a tremendous need to mentor and train the current and next generation of physicians and other clinician groups. Currently, there is little focus on supporting the training of residents and medical students in digital health. To position them well for the digital era, there is a need to teach efficient practices, such as concise documentation in the EHR, which can minimize administrative burden. Moreover, as digital tools continue to gain prominence, it is important to look for ways to implement appropriate guidelines for the use of these tools. The lack of governance structures and guidelines can lead to ill-informed decisions that contribute to downstream problems including burnout. 21 Lastly, as digital tools continue to become a critical aspect of the Canadian health care environment, engagement and continued input from frontline physicians, other clinicians, and patients will only grow in importance. Particularly, clinical engagement will ensure that clinicians can advocate for the ease of a workflow and its effect on administrative burden, as well as the importance of having these aspects become mandatory criteria when evaluating and implementing digital health tools. Thus, exploring ways to have dedicated resourcing and remuneration to support the long-term engagement of physicians in digital health initiatives will be essential.

## Limitations

One limitation of this study pertains to the use of an environmental scan for the retrieval of national results. Given that we only studied gray literature, we may have missed results and findings that are currently available in academic literature. For this reason, our recommendations may have been different had we considered utilizing a combined literature review and environmental scan. In particular, we feel that our recommendations on funding and educational requirements may have been altered with the addition of academic publications.

## Conclusion

As Canada's health care system continues to grapple with workforce shortages and increasing demands, integrating digital health solutions including AI tools will be critical to enhancing physician well-being and efficiencies of practice. This article assesses the current landscape and presents five key system-level recommendations to build the necessary infrastructure for promoting physician wellness related to digital health tools. By carefully evaluating existing wellness initiatives and implementing strategic standardized recommendations, we can work toward fostering a more sustainable and responsible environment for health care professionals with digital health and AI technologies. Future research should focus on identifying actionable recommendations to address physician and patient comfort and trust in the utilization of digital tools as a regular component of health care delivery. This will be particularly important when considering the rapid advancements in AI solutions and the need for further guidelines surrounding legal and privacy policies that health systems should have in place prior to implementing these tools.

## Clinical Relevance Statement

This paper emphasizes the clinical relevance of integrating digital health tools, particularly AI solutions, to support physician wellness. Ongoing challenges such as physician burnout caused by administrative burdens and inefficient digital workflows highlight a need for standardized training, governance, and infrastructure to enhance digital tool adoption to overcome existing burdens. Health care organizations must develop robust strategies, including dedicated committees and national funding initiatives, to ensure equitable access to digital health tools that can improve physician well-being and optimize clinical practice and patient care.

## Multiple-Choice Questions

1. Which of the options below includes the recommendations discussed in this paper to enable the value of digital health, including AI-related wellness initiatives, to be achieved at the national level?
  - Encourage physicians to use any digital tools they want as long as they feel it will improve their wellness and reduce burnout.
  - Hospitals should develop their own digital tools instead of working with digital health vendors.
  - Establish a national funding strategy, standardized toolkits, and implement a comprehensive digital curriculum.
  - Stop implementing digital tools for physician wellness and focus on nondigital solutions that have been used in the past.
  **Correct Answer** : The correct answer is option c. This answer is correct as it was identified in the recommendations section of this paper as the ways to enable the value of digital health, including artificial intelligence-related wellness initiatives. These recommendations are intended to be implemented at the national level.
2. As described in this paper, how can the responsible adoption of digital health in Canadian health care settings be achieved?
  - Establish accountable infrastructures that ensure fair renumeration, robust policy and governance, and enhanced digital literacy for physicians.
  - Only adopt digital health solutions that have been implemented in other hospitals within the nation.
  - When implementing a new digital tool, ensure it is done for all clinician groups at once.
  - Do not involve physicians or other clinical groups in the implementation and adoption process, instead focus on the technical and leadership teams.
  **Correct Answer** : The correct answer is option a. This answer was described in the paper and identified as a way for responsible adoption of digital health in Canadian health care settings.
